# A Reminder That Stress Echocardiography Is Useful in Diagnosing Myocardial Ischemia in Nonobstructive Coronary Artery Disease: Case Series

**DOI:** 10.7759/cureus.17763

**Published:** 2021-09-06

**Authors:** Paramdeep Baweja, Michael J Sweeney, Angel López-Candales

**Affiliations:** 1 Cardiovascular Medicine, University of Missouri Kansas City, Kansas City, USA

**Keywords:** dobutamine stress echocardiography, coronary artery angiogram, microvascular angina, anginal chest pain, cardiac stress test

## Abstract

Identification of ischemia remains critical when assessing individuals presenting with atypical symptoms or in patients with known coronary artery disease (CAD). Several imaging modalities are currently available to attain this diagnostic goal. Unfortunately, not all case presentations are straightforward, particularly when microvascular dysfunction (MVD) is the cause of symptoms in the absence of identifiable epicardial luminal stenosis. Specifically, in such cases, current imaging guidelines do not include stress echocardiography (SE) as a recommended tool when assessing these patients. We present three cases that highlight the utility of SE for identifying MVD and provide mechanistic explanations. We believe that SE should not be completely discarded as an inadequate testing modality; we highlight the potential utility of this imaging modality not only in diagnosing CAD and pre-surgical evaluation of patients but also in identifying patients with MVD.

## Introduction

Stress echocardiography (SE) has not only become a dependable and reproducible imaging tool but also a very cost-effective modality for both the diagnosis and risk stratification of patients with either suspected or known coronary artery disease (CAD) [1]. Reported sensitivity and specificity for dobutamine and exercise SE range from 76-83% and 82-86% vs. 80-85% and 80-88%, respectively [2]. Unfortunately, despite this well-recognized specificity, a high number of false-positive SE continues to be reported and these patients would eventually undergo coronary angiography (CA) without the identification of obstructive CAD [3]. Aside from the reader's over-interpretation, the presence of well-known confounders such as complete left bundle branch block, hypertensive blood pressure response at the time of the test, cardiomyopathic responses, epicardial coronary spasm, or small vessel disease have been proposed as plausible explanations for these false-positive results [3].

Based on our current understanding of the ischemic pathophysiological cascade, stress-induced wall motion abnormalities identified during SE are a very early sign in CAD, which are typically absent in microvascular disease (MVD), despite a comparable reduction in coronary flow reserve [4]. In cases with a concern for possible small vessel disease involvement, the infusion of a vasodilator or the utilization of myocardial contrast echocardiography are the preferred echo modalities [4]. Furthermore, SE is not recommended as part of current imaging guidelines when assessing patients with MVD [5].

In this report, we present three cases that were referred for diagnostic CA after having an abnormal SE with less than hyperdynamic wall motion response along with complaints of chest pain and ischemic ECG changes but unremarkable left heart catheterization findings. We also engage in a review of the literature on this topic.

## Case presentation

Case 1

The first case was that of a 46-year-old female with a past medical history remarkable for Fabry disease, hypertension, chronic complex migraines, obesity, obstructive sleep apnea, and peripheral arterial disease; the patient also had a smoking habit. This patient was initially seen for a regular follow-up appointment and was complaining of chest pain described as tightness, radiating to her left arm, and worse during the evenings and with exertion. She also described a tightening sensation in her neck that occurred with exertion.

**Figure 1:**
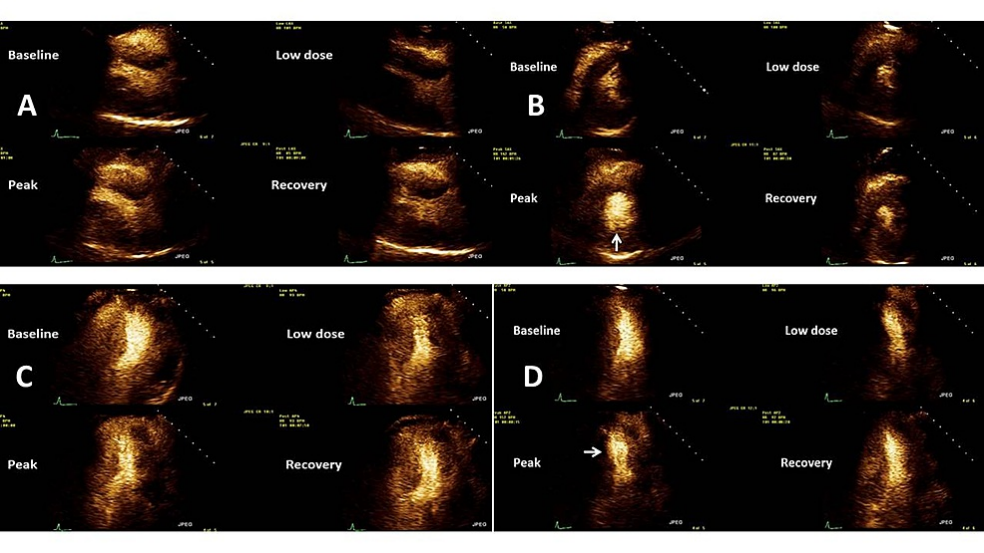
Stress echocardiogram from clinical case 1 (A) Standard parasternal long-axis views of the left ventricle showing baseline, low dose, and peak dose followed by recovery images. (B) Standard short-axis views of the left ventricle showing the same test phases as in the previous. In this set, please note that the arrow demonstrates a lack of contractility of the inferior wall in this view. Inferior wall contractility improves in the recovery images. (C) Standard four-chamber apical views of the left ventricle showing the same test phases as in the previous. (D) Standard two-chamber apical views of the left ventricle showing the same test phases as in the previous. Once again, in this set, please note that the arrow demonstrates a lack of contractility of the inferior wall. Once again, inferior wall contractility does improve in the recovery images

She was referred for a dobutamine stress echocardiogram (DSE) based on these symptoms. No wall motion abnormalities were observed, and there was normal left ventricular systolic function (55-60%) at rest. At peak stress, new mild hypokinesis was seen along the inferior wall, as seen in Figures 1A-1D, suggestive of right coronary artery ischemia. Stress-induced ischemic electrocardiographic changes with chest pain were noted. At this time, she was started on long-acting nitrates for her chest pain and continued on aspirin and statin therapy. Verapamil was discontinued for symptomatic bradycardia.

**Figure 2:**
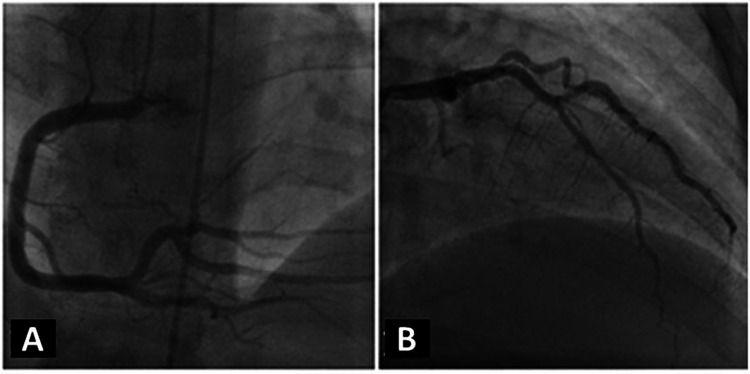
Coronary angiogram from clinical case 1 (A) Standard angiographic view of the right coronary artery and (B) the left coronary artery system from clinical case 1

On CA, it was a right-dominant system with only minor luminal irregularities along a large left anterior descending artery, 20% stenosis of the proximal left circumflex being a medium caliber vessel, and only minor luminal irregularities of a large caliber right coronary artery with no evidence of any other significant disease of the remaining minor arteries (Figures 2A-2B).

The patient was seen two months after her CA and was still complaining of chest pain occurring daily with exertion, with the discomfort lasting up to 20 minutes. She also reported shortness of breath with exertion and palpitations in the week prior. She had been fully compliant with her bilevel positive airway pressure (BiPAP) treatments. Furthermore, she had been physically active, walking one block and climbing up two flights of stairs without any problems. Unfortunately, she was still smoking 10-15 cigarettes per day. She was not consuming alcohol but was smoking marijuana daily. In light of her symptoms, both extended-release metoprolol 25 mg and nifedipine 30 mg were started.

Unfortunately, five days after that follow-up appointment, we received a note stating that the patient had passed away at another hospital facility. No foul play or signs of trauma had been reported. She had been apparently taken to the emergency department due to choking, but there had been no food or anything else lodged in her throat. Therefore, her death was certified as occurring from natural causes.

Case 2

The second case was that of a 67-year-old female with a past medical history of left breast cancer status post-chemotherapy with trastuzumab/pertuzumab and radiation therapy who complained of shortness of breath and chest pain at the follow-up.

**Figure 3:**
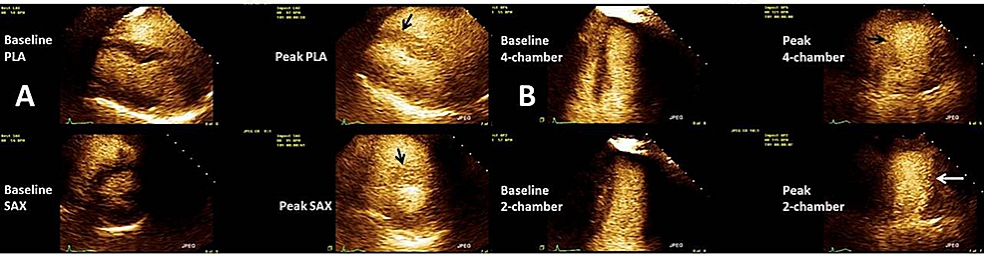
Stress echocardiogram from clinical case 2 (A) Standard parasternal long- and short-axis views of the left ventricle both at rest and post-exercise using a treadmill stress echo protocol. Please note that the black arrows demonstrate the lack of contractility of the anteroseptal and anterior walls at peak exercise. (B) Standard four- and two-chamber apical views of the left ventricle showing the same test phases as in the previous. Please note that the black arrow demonstrates the lack of contractility of the distal inferoseptal wall. The white arrow shows anterior wall ischemia at peak exercise

She was referred for a treadmill SE. At baseline, no wall motion abnormalities were observed, and there was normal left ventricular systolic function (55-60%). At peak stress, new inferior septal, mid anterior, and inferior wall hypokinesis suggestive of ischemia were noted, as seen in Figures 3A-3B. In addition, the patient’s left ventricular ejection fraction (LVEF) dropped from 55-60% to 40% and then returned to its baseline in the recovery period. She had chest pain with ECG changes suggestive of ischemia.

**Figure 4:**
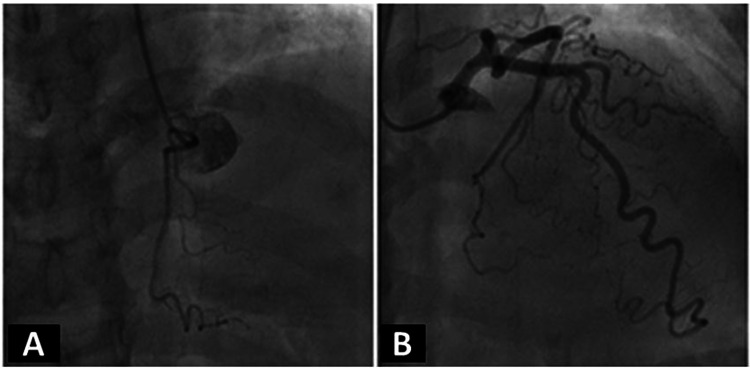
Coronary angiogram from clinical case 2 (A) Standard angiographic view of the right coronary artery and (B) the left coronary artery system from clinical case 2

Based on these results, the patient was referred for CA, which showed no evidence of significant disease in the left main or left anterior descending, both being large-caliber vessels, no evidence of significant disease on a medium caliber-sized left circumflex, and no disease of a very small non-dominant right coronary artery (Figures 4A-4B).

She was later seen at the follow-up and some adjustments were made to her continuous positive airway pressure (CPAP) therapy with complete resolution of her chest pain, and she was reported to be sleeping better. She was treated with aspirin, statin, lisinopril for blood pressure control and was also prescribed sublingual nitrates to be used if necessary; however, she stated that she did not need to use nitrates anymore.

Case 3

The third case involved a 49-year-old male with a medical history of hypertension, obstructive sleep apnea, and thrombotic thrombocytopenic purpura. This patient was an active smoker and was homeless at the time of testing. He endorsed complaints of atypical chest pain but did not provide an accurate history. Compliance with prescribed medications was somewhat questionable and his exercise tolerance was difficult to assess. He was not able to walk on the treadmill. Therefore, we proceeded with a DSE protocol as he was being evaluated for elective cholecystectomy. Baseline images showed no wall motion abnormalities with normal left ventricular systolic function (LVEF: 55-60%).

**Figure 5:**
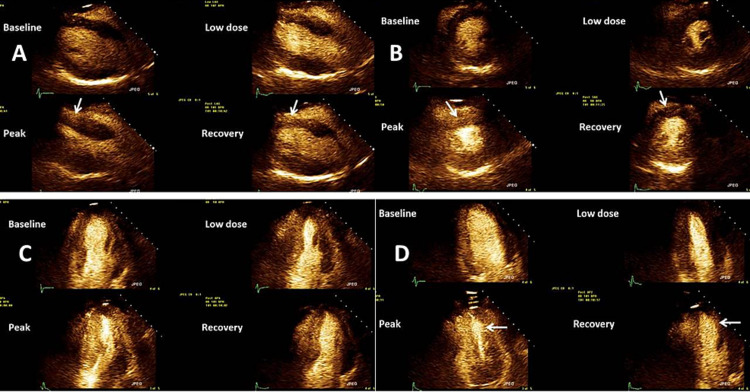
Stress echocardiogram from clinical case 3 (A) Standard parasternal long-axis views of the left ventricle showing baseline, low-dose, and peak-dose followed by recovery images. Please note that the white arrow shows a lack of contractility of the anteroseptal wall. Contractility does not improve in the recovery images. (B) Standard short-axis views of the left ventricle showing the same test phases as in the previous. Please note that the white arrow shows a lack of contractility of the anterior wall and contractility does not improve completely in the recovery images. (C) Standard four-chamber apical views of the left ventricle showing the same test phases as in the previous. (D) Standard two-chamber apical views of the left ventricle showing the same test phases as in the previous. Please note that the white arrow shows a lack of contractility of the anterior wall and contractility does not seem to improve in the recovery images

**Figure 6:**
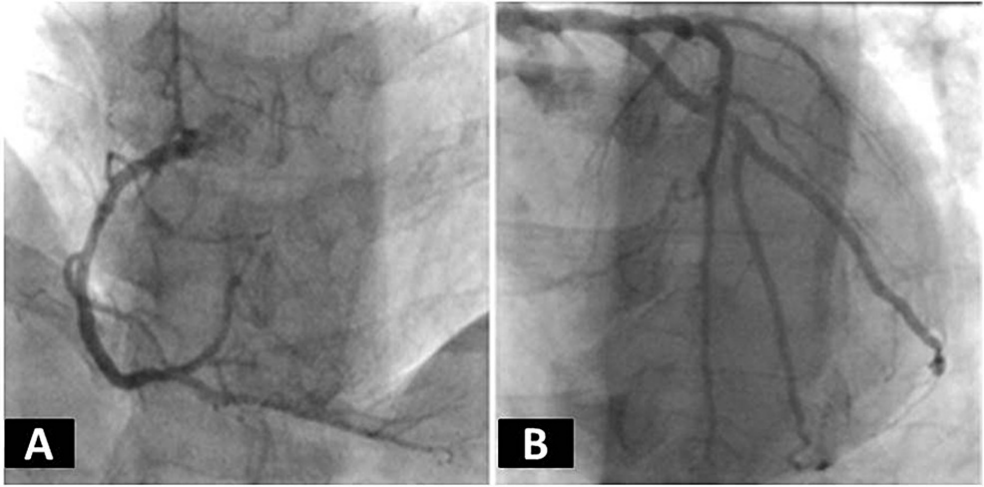
Coronary angiogram from clinical case 3 (A) Standard angiographic view of the right coronary artery and (B) the left coronary artery system from clinical case 3

During his DSE, new anteroseptal and anterior wall abnormalities were seen both at peak and during recovery, as seen in Figures 5A-5D, with transient ischemic ECG changes and chest pain during the infusion of dobutamine, which both resolved later. Given his social situation, he was admitted for diagnostic CA. No epicardial luminal disease was noted (Figures 6A-6B), and the patient was subsequently discharged.

## Discussion

Recognition of MVD or ischemia without obstructive coronary disease [ischemia of non-occlusive coronary artery disease (INOCA)] has become increasingly important as more patients are being diagnosed, particularly women; moreover, this clinical entity may play a crucial role in the pathophysiological mechanisms of heart failure with preserved ejection fraction and Takotsubo cardiomyopathy [6,7]. Surely, even though our understanding of MVD coronary disease has certainly improved, we still recognize the need for better, more reliable, and reproducible measurements that will aid in improving prognostication and treatment plans [8]. This is clearly relevant, particularly when MVD and the resultant perception of angina not only represents a huge diagnostic dilemma but also creates a significant degree of uncertainty with regard to diagnosis, particularly when adverse events are known to occur in both female and male patients [9,10]. In addition, the diagnosis can become challenging since MVD can exist in the setting of both obstructive and nonobstructive coronary disease [8-10]. Even in the absence of a clearly defined definitive pathophysiologic basis, a combination of factors including endothelial dysfunction, coronary spasm, inflammation, and atherosclerosis can all disrupt myocardial blood flow by combining potentially structurally abnormal atherosclerotic coronary arteries with vasomotor disorders causing dynamic coronary arteriolar alterations [3,5].

Based on these principles, current tools used in the assessment of coronary MVD include the administration of vasodilating medications such as adenosine for the measurement of coronary flow reserve invasively; assessment of myocardial perfusion reserve using contrast echocardiography, Doppler echocardiography, or CT perfusion; cardiac MRI or positron emission tomography (PET) [5,10]. However, of these, PET is associated with the most prognostic data at present and it would be considered the test of choice [5,10]. Obviously, factors such as expertise, radiation exposure, cost concerns, and limited availability dictate which test is finally performed.

Even when SE has lagged behind the more technologically advanced imaging modalities, this noninvasive tool is still used widely. Although it is difficult to determine the current trends in SE use, data published by Kini et al. using administrative claims from members aged 25-64 years belonging to a large, nationally managed care company from January 2005 through December 2012 showed decreased rates of nuclear stress testing while the use of stress echo was found to have increased. Surely, these results, aside from being driven by unique characteristics of populations and the health system involved, do not truly reflect the national trends with regard to the real use of SE [11].

Echocardiography has definitely undergone a rebirth with the introduction of myocardial contrast perfusion imaging [12-15]. However, its utility has been limited to places with such expertise and resources to perform this test, which has undoubtedly curtailed its clinical utility as most studies using contrast perfusion have originated in the research setting. Therefore, even when these newly added imaging modalities are unavailable, SE still remains mostly useful when assessing low-risk chest pain cases [16]. Furthermore, SE not only should be routinely considered as valuable for routine assessment of either chest pain or for preoperative evaluations, but it also might be useful in identifying MVD, particularly if no other imaging options are available, as clearly stated in the latest 2019 European Society of Cardiology (ESC) guidelines [17].

It is our belief that the lack of either myocardial perfusion or strain imaging during SE could still be valuable for identifying MVD [13,18], particularly if a less than hyperdynamic wall contractility response is seen at peak stress. In fact, we should be mindful that a hyperdynamic response was the original definition of a normal response to stress and it should remain the basis of all our SE interpretations [9,10]. Certainly, many would undoubtedly argue that this subjective interpretation is operator-dependent as it truly appears to be an interpretation that is not derived or determined by any objective means.

We believe it is important to address the following pertinent elements with regard to our patients in these clinical cases. Firstly, regarding the case of the patient with Fabry disease, life expectancy in these patients is known to be reduced for both males and females [19]. In fact, cardiac causes are the second most common cause of death in these patients [19]. Furthermore, cardiac microvascular function abnormalities in Fabry patients have been demonstrated by measurements of myocardial blood flow and coronary flow reserve, an index of microvascular function [19]. More relevant to our case series, a survey of female Fabry patients has revealed that cardiac ischemia could be confirmed by ECG and serological markers in the absence of coronary artery stenosis, suggesting that ischemia in these patients was of microvascular origin. Secondly, one of the effects of chemotherapy occurs at the vasculature level and may cause MVD [20]. The latter not only has been shown to be an early indicator of numerous cardiovascular disease phenotypes but is also enhanced by the presence of traditional cardiovascular risk factors [20]. So in our case, the effect of previous chemotherapy could have resulted in the presence of MVD and explain the patient’s symptoms and stress echo results. Thirdly, pertinent factors regarding smoking should also be considered as the reviewer suggests. Although coronary artery spasm was first reported in 1993 [21], none of our patients showed signs or presentations of coronary artery spasm. Furthermore, although cigarette smoking is a recognized independent risk factor for cardiovascular disease, studies have shown controversial results with regard to mortality rates and prognosis of patients after reperfusion with percutaneous coronary intervention [22]. To that end, cardiac MRI data have suggested that smoking does not display any significant association with either microvascular obstruction or index of microvascular resistance. Some might argue that the number of false-positive and negatives during SE continue to be an issue. Regardless of the respectable high sensitivity and specificity of SE in terms of its diagnostic accuracy, a subset of patients with false-positive tests (defined as <50% diameter stenosis on subsequent CA) is a well-documented reality. However, our claim of diagnostic accuracy was based on when a hyperdynamic wall motion response is identified in the presence of symptoms and/or ECG changes. The latter might certainly reconcile new data that has suggested that the presence of positive electrocardiographic findings in the presence of a normal SE imaging has identified a population of patients who are at slightly increased risk of adverse cardiac events.

In summary, SE is one of the most utilized imaging tools for the identification of obstructive epicardial CAD. Recent advances in both imaging acquisition and stress protocols have enabled the use of this testing modality for the assessment of myocardial viability, efficacy of anti-ischemic medical therapy or percutaneous interventions in patients with known CAD, and the evaluation and management of valvular heart disease, as well as in a number of non-ischemic conditions to assess hemodynamic parameters. Most importantly, SE, aside from being comparable in terms of accuracy when compared to other modalities such as single-photon emission CT, is the least expensive testing modality; it is widely available, and it does not expose patients to radiation.

However, the utility of SE in terms of MVD has been limited as this clinical entity has required the identification of a reduction in coronary flow reserve. Traditionally, this has been accomplished during invasive testing by the identification of regional perfusion changes rather than wall motion abnormalities during stress. Our clinical case series highlights the possibility of using SE for the assessment of MVD. We believe that if the expected hyperdynamic wall motion response is not seen in patients with symptoms or ECG changes consistent with ischemia, the possibility could be entertained if a CA fails to disclose significant obstructive CAD.

It is also important to keep in mind that even though we only provide evidence of three clinical cases showing the presence of ischemic symptoms and ECG changes with a lack of a hyperdynamic wall motion response at peak stress in the absence of obstructive coronary artery luminal disease suggesting the possibility of MVD, the presence of a normal CA does not exclude diffuse atherosclerosis with abnormal epicardial resistance or atherosclerosis with positive remodeling.

The diagnosis of MVD is important because of its therapeutic and prognostic implications and due to the fact that the number of patients with coronary MVD is increasing. This case presentation directs the attention of SE-reading physicians to the notion that a lack of a hyperdynamic response in terms of wall motion should be carefully evaluated and recognized.

Therefore, SE should not be completely discarded as an inadequate testing modality, especially given that new and grandiose technologically advanced imaging modalities are not widely available. Furthermore, SE should not be completely relegated for the sole use of assessing low-risk chest pain patients presenting to the emergency department.

## Conclusions

As discussed in our case presentations, SE remains a very valuable imaging tool that not only plays a crucial role in diagnosing CAD and the pre-surgical evaluation of patients but is also useful for identifying patients with MVD or INOCA. It simply requires a good eye and careful interpretation.
